# Nutrient Intake and Food Habits of Soccer Players: Analyzing the Correlates of Eating Practice

**DOI:** 10.3390/nu6072697

**Published:** 2014-07-18

**Authors:** Pablo M. García-Rovés, Pedro García-Zapico, Ángeles M. Patterson, Eduardo Iglesias-Gutiérrez

**Affiliations:** Department of Functional Biology, Area of Physiology, University of Oviedo, Oviedo 33006, Spain; E-Mails: pgarciar@clinic.ub.es (P.M.G.-R.); pzapico@terra.com (P.G.-Z.); patterson@uniovi.es (A.M.P.)

**Keywords:** energy and macronutrient intake, soccer, eating practice, nutrition education

## Abstract

Despite the impact and popularity of soccer, and the growing field of soccer-related scientific research, little attention has been devoted to the nutritional intake and eating habits of soccer players. Moreover, the few studies that have addressed this issue suggest that the nutritional intake of soccer players is inadequate, underscoring the need for better adherence to nutritional recommendations and the development and implementation of nutrition education programs. The objective of these programs would be to promote healthy eating habits for male and female soccer players of all ages to optimize performance and provide health benefits that last beyond the end of a player’s career. To date, no well-designed nutrition education program has been implemented for soccer players. The design and implementation of such an intervention requires *a priori* knowledge of nutritional intake and other correlates of food selection, such as food preferences and the influence of field position on nutrient intake, as well as detailed analysis of nutritional intake on match days, on which little data is available. Our aim is to provide an up-to-date overview of the nutritional intake, eating habits, and correlates of eating practice of soccer players.

## 1. Introduction

Soccer is currently the world’s most popular sport, and according to the 2006 Big Count FIFA survey, is played by over 265 million people worldwide, 10% of whom are female. It is also an undeniable sociological and media phenomenon. During the last FIFA World Cup held in South Africa in 2010, a total of 71,867 h of soccer were broadcast to a cumulative audience of over 3.2 billion people around the world, according to the 2010 FIFA World Cup South Africa™ Television Audience Report.

Recent decades have seen major advances in the field of sports sciences, particularly in soccer. Most soccer-related biomedical research has focused on three main areas: the physiological demands of the game [1,2,3,4,5,6,7]; the anthropometric characteristics and physiological and performance capabilities of the players [8,9,10,11,12,13,14,15]; and the use and bioavailability of energy substrates during training and match-play [1,16,17,18]. A number of studies have also analyzed the influence of playing position on these parameters [1,5,7,12,14,19,20,21,22,23,24,25,26,27,28,29,30].

Based on the results of these studies, specific nutritional recommendations have been developed for soccer players. The objectives of these guidelines are to optimize performance during training and competition, improve and accelerate recovery, achieve and maintain an optimal body weight and physical condition, and minimize the risk of injury and illness [31,32,33,34,35,36,37]. Recommended macronutrient intake has traditionally been expressed as a percentage of total energy intake (% energy). However, total energy intake is a confounding variable that can lead to misinterpretation of results obtained in terms of absolute macronutrient intake, hindering comparisons between studies. Nowadays, the use of % energy is wholly discouraged for carbohydrates (CHO) and proteins, the intake of which is preferably expressed in g/kg BM/day. Although these two forms of expressing macronutrient intake are not equivalent, they are complementary. We have found that the simultaneous use of both options is the best way to provide a complete overview of the macronutrient intake of soccer players.

However, the majority of studies conducted have revealed inadequate nutritional intake in both male and female soccer players [38,39,40,41,42,43,44,45,46,47], underscoring the need to improve short and long-term adherence to these recommendations. While there is unanimous agreement on the need to design and implement nutrition-specific intervention programs for soccer players, there is a paucity of information on the eating habits and correlates of nutritional behavior of soccer players. Understanding the influence of these factors is essential for the design and implementation of effective dietary and nutrition education programs, in order to optimize performance and health through nutrient intake. Here, we provide an up-to-date overview of the nutritional intake, eating habits, and correlates of eating practice of soccer players.

## 2. Methods: Search Strategy

Several databases were used in searching articles about the nutritional intake of soccer players published up to December 2013: PubMed (US National Library of Medicine National Institutes of Health), Scopus, and Science Direct. Various combinations of the following keywords were used: nutrition, intake, expenditure, energy, carbohydrates, proteins, lipids, fat, soccer, football, female, education, intervention, food preferences, playing position, playing role, and match. We searched the bibliographies of the retrieved articles in order to identify additional articles.

Among the articles selected, only were included in this revision those that: (a) were published from year 2000 onwards; (b) included players at the age of 16 or over, which is the minimum age to be competent to sign a professional contract in most countries; (c) assessed dietary intake by weighed food records or estimated food records (using household measures); (d) included data from at least three days of dietary recording.

Considering all the above, a total of 28 articles were identified and 13 met the general review inclusion criteria.

## 3. Nutritional Intake and Eating Habits of Soccer Players

Despite the popularity of soccer and the burgeoning field of soccer-related scientific research, the nutritional intake of soccer players has attracted surprisingly little research attention. The few studies that have addressed this issue are summarized in Table 1 (male players) and Table 2 (female players). Several authors have also analyzed the intake and nutritional status of vitamins and minerals [40,41,42,48], but these articles were not included in this revision due to the methodological difficulties and limitations for an accurate assessment and interpretation of micronutrient intake data [49].

In the following sections, we will discuss the reasons for the significant variability in energy and macronutrient intake of soccer players, as well as gender differences in nutritional behavior.

### 3.1. Energy Intake and Expenditure

Few studies have analyzed energy balance in soccer players, and only two have met the inclusion criteria for this review [44,47]. Other studies conducted in this area have produced highly variable results, possibly due to the use of diverse methodologies and experimental designs, and to differences in the ages, competitive levels, and training loads of the players studied. Several authors have focused on the assessment of total daily energy expenditure (EE), but provide no information about nutrient intake, and thus are not included in Table 1 or Table 2. In these studies, the techniques used to measure EE included the doubly-labeled water method [50] and indirect calorimetry (resting EE) [51]. Although accurate [34,52], these are complex and expensive techniques, and are of limited value with large groups or for routine use. Some studies have evaluated energy expenditure during match-play or training without calculating total daily energy expenditure [1,6,53,54], and hence have not been included in this review.

**Table 1:** Studies analyzing the nutritional intake of male soccer players.

Study characteristics and Nutrients	Ruiz *et al.* 2005 [46]		Garrido *et al.* 2007 [41]		Caccialanza *et al.* 2007 [39]		Rusell and Pennock 2011 [47] ^b^	Iglesias-Gutiérrez *et al*. 2012 [43]
	*Team C*	*Team D*	*Team M*	*Team B*	*Team T0*	*Team T1*		
Team population (*n*)	Junior teams of a Spanish	Junior teams of a Spanish	Junior teams of a Spanish	Junior teams of a Spanish	Junior teams of an Italian		Junior teams of a British	Junior teams of a Spanish
	fourth division team (19)	fourth division team (24)	first division team (33)	first division team (29)	first division team (43)		second division team (10)	first division team (87)
Age (years)	16.6 ± 0.15	20.9 ± 0.47	16.9 ± 1.5	16.1 ± 1.4	16 ± 1	16 ± 1	17 ± 1	18 ± 2
Food record method	Weighed food record		Weighed food record		Estimated food record		Estimated food record	Weighed food record
					(household measures)		(household measures)	
Recording period (days)	3		5		4		7	6
Energy intake (kcal)	3478 ± 223	3030 ± 141	2740 ± 531	3148 ± 619	2560 ± 636	2640 ± 614	2831 (164)	2796.4 ± 525.8
*Proteins*								
g	150.2 ± 5.1	132.8 ± 6.3	111 ± 23	114 ± 22	101 ± 23	104 ± 28	114 (8)	119 ± 24
g/kg BM	2.03 ±0.2	1.81 ± 0.1	1.5 ± 0.3	1.6 ± 0.3	1.5 ± 0.4	1.5 ± 0.4	1.7 (0.1)	1.6 ± 0.4
% energy	16.9 ^a^	17.7 ^a^	14.7 ^a^	16.3 ^a^	16.6 ± 2.1	17.0 ± 2.4	16 (1)	17 ± 2
*CHO*								
g	392 ± 37	334 ± 16	316 ± 70	392 ± 85	339 ± 89	352 ± 86	393 (18)	338 ± 70
g/kg BM	5.32 ± 0.4	4.57 ± 0.2	4.4 ± 1.1	5.6 ± 1.4	4.9 ± 1.5	5.0 ± 1.3	5.9 (0.4)	4.7 ± 1.1
% energy	45.2 ^a^	44.6 ^a^	49.5 ^a^	46.1 ^a^	52.9 ± 4.0	53.4 ± 5.5	56 (1)	45 ± 5
*Lipids*								
g	154 ± 4.9	128 ± 9.8	-	-	87.0 ± 25.0	87.1 ± 26.4	100 (9)	116 ± 30
% energy	38.4 ^a^	38 ^a^	35.7 ^a^	37.5 ^a^	30.5 ± 5.5	29.6 ± 4.3	31 (1)	37 ± 5
MUFA (g)	59.5 ± 2.7	50.4 ± 4.5	38.2 ± 10.1	44.5 ± 9.4	-	-	32 (8)	-
PUFA (g)	33.4 ± 2.7	19.9 ± 2.5	17.7 ± 5.8	20.4 ± 5.2	-	-	17 (5)	-
SFA (g)	44.3 ± 2.7	36.4 ± 3.9	44.7 ± 12.9	49.3 ± 14.3	-	-	40 (16)	-

BM: Body mass; MUFA: Monounsaturated fatty acids; PUFA: Polyunsaturated fatty acids; SFA: Saturated fatty acids. Unless stated otherwise, data are presented as Mean ± Standard deviation: ^a^ Mean; ^b^ Mean (Standard error of the mean). All data about energy intake have been converted to kcal. Blank cells indicate that this information has not been provided by the author(s).

**Table 2:** Studies analyzing the nutritional intake of female soccer players.

Study characteristics and Nutrients	Mullinix *et al.* 2003 [45]	Clark *et al.* 2003 [40]		Abood *et al.* 2004 [38]	Martin *et al.* 2006 [44]	Gravina *et al.* 2012 [42] ^1^
		*Pre-Season*	*Post-Season*			
Team population (*n*)	U-21 U.S. women’s National	American NCAA Division I (13)		American NCAA Division I (15)	English international	Spanish First and
	soccer team (11)				Players (16)	Second division (28)
Age (years)	19.2 ± 1.1	19.7 ± 0.7	20.0 ± 0.9	19.6 ± 1.0	25.5 ± 3.9	21 ± 6
Food record method	Estimated food record	Estimated food record		Estimated food record	Estimated food record	Weighed food record
	(household measures)	(household measures)		(household measures)	(household measures)	
Recording period (days)	3	3	3	3	7	8
Energy intake (kcal)	2015 ± 19	2290 ± 310	2291 ± 310	1969 ± 414	1904 ± 366	2271 ± 571
*Proteins*						
g	79 ± 33	87 ± 19	59 ± 17	-	-	-
g/kg BM		1.4 ± 0.3	1.0 ± 0.3	-	1.2 ± 0.3	-
% energy	15 ^a^	15 ± 3	13 ± 2	13 ± 2	16.8 ± 2.1	15 ± 2
*CHO*						
g	282 ± 118	320 ± 70	263 ± 71	-	-	-
g/kg BM	-	5.2 ± 1.1	4.3 ± 1.2	-	4.1 ± 1.0	-
% energy	55 ^a^	55 ± 8	57 ± 7	59 ± 9	53.8 ± 6.8	-
*Lipids*						
g	67 ± 28	75 ± 13	66 ± 29	-	-	-
% energy	30 ^a^	29 ± 6	31 ± 7	24 ± 7	28.8 ± 6.6	37 ± 7
MUFA	15 ± 8	-	-	-	-	-
PUFA	8 ± 6	-	-	-	-	-
SFA	22 ± 10	-	-	-	-	12.4 ± 3

BM: Body mass; MUFA: Monounsaturated fatty acids; PUFA: Polyunsaturated fatty acids; SFA: Saturated fatty acids. Unless stated otherwise, data are presented as Mean ± Standard deviation: ^a^ Mean. Unless stated otherwise, MUFA, PUFA, and SFA intake are referred as g/day: ^1^ intakes expressed as percentage of total energy intake. Blank cells indicate that this information has not been provided by the author(s).

Most authors measure energy and nutrient intake using food records (weighed or estimated), although others provide only vague details of the methods used (“food recall”, “dietary record”). Only one study by Noda and coworkers [48] of Japanese collegiate players used a food frequency questionnaire, the validity of which is limited for the estimation of energy and nutrient intake [55]. Thus, in order to avoid the influence of methodology, only studies conducted using weighed food records or estimated food records (using household measures) were included in this revision. Furthermore, studies of male and female athletes frequently highlight the possibility of underreporting (intentional or unintentional) due to both under-recording and under-eating, even when weighed records are used [55,56]. Achieving sufficient energy is essential to maintain lean mass, to maximize the benefits of training sessions, and to ensure an adequate intake of all nutrients [57]. An accurate estimation of energy intake is thus crucial in planning a successful nutritional strategy.

Given these methodological considerations, we will next focus on the results obtained in different studies.

As expected, the energy expenditure estimated by Russell and Pennock [47] for male players (3618 (61) kcal) is higher than their female counterparts (2154 ± 596 kcal), estimated by Martin *et al.* [44]. Although no generalization is possible in this situation, these discrepancies may be explained by gender differences in training loads and in other parameters such as body composition [34,57]. Furthermore, these studies used physical activity questionnaires to estimate EE. However, this approach is limited in its validity and reliability [39,57,58,59].

While there is great variability in the values reported for male players between studies, the typical daily energy intake reported is 2500–3100 kcal, although intakes as high as 3478 ± 223 kcal [46] have also been recorded.

Estimates of energy intakes for female players are less variable, ranging from 1904 ± 366 kcal [44] to 2291 ± 310 kcal [40]. These lower intakes compared with male counterparts may be partly due to the aforementioned specific energy needs of female players, as well as underreporting of dietary intake. Studies suggest that women are much more likely to underreport than men [60,61,62,63].

### 3.2. Macronutrient Intake

Given the well-documented importance of nutrition in optimizing performance and health, it is somewhat surprising that the nutritional intake of soccer players, particularly male players, has been systematically described as inadequate. Most studies have reported daily CHO intakes lower than those recommended, while the protein and lipid intake of the majority of players exceeds recommended amounts. The reported macronutrient intake exclusively refers to food sources, since information about the use of supplements was not provided by the authors.

#### 3.2.1. Carbohydrates

Adequate CHO intake is a key nutritional factor required to cope with training demands and to promote recovery between games. Burke and colleagues [34] proposed 5–7 g/kg of body mass (BM)/day as a reasonable target range for CHO intake for moderate training and competitive demands, increasing to 7–10 g/kg BM/day for intensive training or maximal glycogen refueling. No differences in CHO utilization and storage between male and female soccer players have been described [64], suggesting that CHO intake recommendations are valid for both genders. However, the likelihood that total CHO intake is sufficient to optimize glycogen synthesis and utilization is lower in women, due to their lower energy intake [34,64].

All the studies of male players have reported CHO intakes <6 g/kg BM (Table 1), and similar CHO intakes have been reported for female players, ranging from 4.1 g/kg BM [44] to 5.2 g/kg BM [40], despite their lower energy intake (Table 2). However, the contribution of CHO to total energy intake reported in women (>55% in most studies) is greater than in men (<50% in the majority of studies). Given that a contribution of >55% of energy intake from CHO has been traditionally recommended for soccer players [35], these figures suggest that, although the absolute intake of CHO of male and female players is not optimal, the diet of female players is better macronutritionally balanced than that of their male counterparts. This is a very important issue, since not only the amount but also the balance of macronutrients can profoundly affect health [65].

The following question arises: to what extent does this inadequate CHO intake affect performance? There are plenty of scientific studies showing that diets high in CHO or CHO solutions consumed before exercise allow an increase in muscle glycogen concentration, delaying the onset of fatigue and improving performance [66]. Some of them have specifically focused on soccer, finding improvements in total distance covered [67,68], in the ability to perform high-intensity activities [68,69], and in technical performance [70,71,72], together with a reduction in net muscle glycogen utilization throughout the game [73,74]. However, a review by Bangsbo and colleagues [2] found that while most studies reported almost total depletion of muscle glycogen stores by the end of a match, not all muscle fibers show the same degree of depletion. Furthermore, these decreases in muscle glycogen did not always reach levels lower than those required to maintain maximal glycolytic rate. According to Zehnder and colleagues [75], diets providing around 5 g of CHO/kg BM might be enough to replenish muscle glycogen within 24 h of a soccer match. However, cumulative deficits of about 10% in glycogen replenishment might lead to performance impairments. Therefore, while a moderate CHO intake may not reduce the ability of trained athletes to complete rigorous training sessions for up to a month, a high-CHO diet optimizes improvements in performance [76]. A recommended daily nutrient intake that includes large amounts of CHO thus seems reasonable in order to optimize performance and cope with the demands placed on players.

#### 3.2.2. Proteins

Very few studies have specifically evaluated the protein requirements of soccer players. To the best of our knowledge, only two studies have assessed this parameter by determining the nitrogen balance of adolescent male soccer players [31,32]. Those authors concluded that the protein requirements of these adolescents were above the recommended daily allowance of non-active counterparts, and reported a positive nitrogen balance from a mean protein intake of 1.57 g/kg BM. This result is in line with the recommended 1.4–1.7 g/kg BM proposed by Lemon and coworkers [36] for soccer players, based on the results of strength and endurance studies. It is also in accordance with the findings of Tipton and Wolfe [77], who recommended intakes of 1.2–1.7 g/kg BM for athletes. Little is known about gender differences in protein metabolism [64], although it seems reasonable to suggest that this recommendation is valid both for male and female soccer players.

The protein intake of soccer players typically reported in the literature ranges from 1.5 to 1.8 g/kg BM for male players (Table 1) and 1.2–1.4 g/kg BM for females (Table 2). As for CHO, protein intake relative to BM is lower in females due to their lower energy intake. The absolute amount of proteins ingested appears to be adequate in both males and females, albeit slightly higher than recommended for male players in some studies. These findings suggest that emphasis should not only be placed on the amount of proteins ingested, as the recommended intake is easily and spontaneously achieved by most soccer players from a variety of food sources with different amino acid profiles, but also on the timing and quality of protein intake. In this sense, numerous studies have shown that protein ingestion near the time of exercise can promote a positive nitrogen balance across the active muscles and exert a more effective adaptation to training [76,77,78]. Furthermore, although the assessment and definition of protein quality is complex, the evidence available to date also highlights its importance to satisfy the demands for protein synthesis even at high intakes [79,80].

#### 3.2.3. Lipids

It is difficult to estimate the contribution of lipid metabolism in intermittent sports such as soccer. The limited information available indicates that, given the highly aerobic nature of soccer, lipid oxidation is likely very important, especially during periods of rest after high intensity activities during match-play or training [2,16,18]. However, lipid intake recommendations are usually calculated with a view to facilitating adequate CHO intake, and not to contribute to energy metabolism during soccer play [81]. It has been suggested that players should obtain <30% of their total energy intake from fat [82]. However, all the studies of male players have reported lipid intakes >30%, and intakes of 37% or higher are not exceptional (Table 1). This high lipid intake clearly limits the likelihood of achieving an adequate CHO intake. Most studies of female players have reported lipid intakes ranging from 29%–30%. Given their lower energy intake, their absolute fat intake (66–75 g) is considerably lower than that of their male counterparts (typically 100–130 g). These data indicate that close attention should be paid to reducing lipid intake, especially in male players.

Despite the growing importance of the dietary proportion of different fatty acids [83], there is little information available on this parameter in soccer players [38,40,42,44,45]. Furthermore, the few studies conducted express fatty acid intake using g/day, when recommendations for general population are expressed as % of total energy intake, and some contain incomplete data [42]. This hinders comparison with reference values and other studies and makes it impossible to draw firm conclusions, although it appears that the % of total energy intake provided by saturated fatty acids exceeds the recommendations (<10% of total energy intake) in male players, while polyunsaturated fatty acids are far below the recommendations (6%–11% of total energy intake). One reason why PUFA intake could be notably lower in the populations studied is because they over-represent study samples from Spain and Italy, *i.e.*, Mediterranean countries where MUFA intake (predominantly from olive oil) is proportionately higher than in other parts of the world. Since reducing lipid intake is a desirable objective for male players, attention should also be paid to optimizing the intake of the different fatty acids.

### 3.3. Food Intake and Eating Practice

Few studies have investigated the food sources of the nutrients ingested by soccer players. To the best of our knowledge, no information on the food intake of female soccer players is available.

We previously reported that the food intake of young male soccer players is derived from the following food groups: cereals, derivatives, and potatoes; milk and dairy products; meat, poultry, and derivatives; and oil; which together provided 65% of total daily energy intake, with a marginal contribution from vegetables and fruits [43].

Noda and coworkers reported that cereals and derivatives (rice, bread, and noodles); beverages other than water; milk and derivatives; and vegetables, potatoes, and seaweeds accounted for almost 85% of the total amount of food ingested by soccer players (g), but provided no information about the contribution of these food groups to energy or nutrient intake [48]. No information is available on the distribution of food intake between different meals (breakfast, lunch, dinner, snacks) or its relationship with energy and nutrient intake. As mentioned before, the food record method used in this study was a food frequency questionnaire, which is related to the limitations observed.

Similarly, little is known about the distribution of energy and macronutrient intake across the main meals [41,46] (Table 3). No information is available for female players, except for one study by Reed and colleagues [53]; data from this study are not included in Table 3 as surprisingly they were only mentioned but not included in the original manuscript. The disparity in the results obtained for male players is huge. In the study of Garrido and colleagues [41], players were assessed while living at residence halls, where they were provided with set menus for their main meals, and no morning snack was reported, which may account for some of the variation between studies. However, despite these differences, lipids consumed in lunch and dinner significantly contribute to total energy intake. This underscores the need to improve the design of these meals in order to lower their fat content, and to obtain more information about the food sources of nutrients in each individual meal.

In short, these findings indicate the need for the design and implementation of nutrition education programs, especially for male players, as previously stated by other authors working with different groups of soccer players [31,39,40,41,43,44,45,46,84,85,86,87,88]. However, few studies of diet and nutrition in soccer players provide information about the food sources and related behaviors that account for the inadequate nutrient intake observed. This information is crucial, as nutrition-specific education programs should focus on both food and eating practice, not only on nutrients.

## 4. Nutrition Interventions

Although many authors have emphasized the need for nutrition intervention programs to optimize the nutritional intake of soccer players, only one group has actually implemented such a program [38], working with female players. Unfortunately, this study suffers from several important limitations. Pre-intervention nutrient intake was very close to recommended values (protein, 13%; CHO, 59%; and lipids, 24% of total energy intake), suggesting that the intervention was not completely justified, especially when no information about food selection is provided. Moreover, the nutritional intervention conducted failed to improve nutrient intake, although increases in nutrition knowledge and self-efficacy were reported. As this study focused solely on nutrient intake, it is impossible to know whether the intervention had any impact on food intake and eating practice.

**Table 3:** Studies examining the distribution of energy intake (%) across different meals and the proportion of energy intake (%) provided by the different macronutrients at each meal.

Meals and Nutrients	Ruiz *et al.* 2005 [46]		Garrido *et al.* 2007 [41]	
	Team C	Team D	Team M	Team B
Breakfast	19.9	10.7	24.9	22.1
Protein	10.9	14.4	10	12
CHO	50.3	35.6	61	60
Lipids	38.8	50.0	29	28
Morning snack	5.28	2.36	-	-
Protein	6.82	16.2	-	-
CHO	52.7	35.0	-	-
Lipids	40.4	49.1	-	-
Lunch	36.8	44.7	27.5	34.5
Protein	20.5	19.5	18	20
CHO	36.8	34.0	39	41
Lipids	42.7	46.4	43	39
Afternoon snack	11.5	6.63	24.7	16.2
Protein	11.6	10.6	11	9
CHO	41.4	46.2	53	52
Lipids	47.0	43.3	36	39
Dinner	33.4	33.7	22.8	26.4
Protein	19.9	18.6	18	19
CHO	32.2	36.3	45	39
Lipids	47.9	45.1	34	42

CHO: carbohydrates. Data are presented as Mean. Blank cells indicate that this information was not provided by the authors.

Working with a cohort of male players, Bangsbo designed a nutritional intervention program in which 60% of the daily diet was controlled, while the remaining 40% could be chosen by the players, according to general guidelines provided by the researchers [89]. While this intervention resulted in an increase in CHO intake from 45% to 65% of energy consumed, it cannot be considered an educational nutritional intervention. Interestingly, however, the foods selected to improve the CHO content of the diet are easily accessible and commonly found in most households and menus, suggesting that there is no need for a drastic change in food habits to achieve a better nutritional intake. However, as shown in Table 1, the nutritional intake of players assessed while living at residence halls [41], where set menus are offered, does not differ greatly from that of players assessed in their home environment. Thus, controlling the menus offered to the players does not appear to be a very effective or practical means of optimizing their intake or constitute an alternative to the implementation of nutrition education programs.

Research by Ono and colleagues provided a unique and interesting perspective on the cultural correlates of the food and nutritional intake of Premier League soccer players [90]. Their study sought to identify the underlying reasons for the inadequate intake of players; while the food intake of the players assessed was recorded, these data were not provided in the manuscript, and hence are not included in Table 1. The authors concluded that attention to nutrition is rarely a club priority, and found that clubs tend not to work with sports nutritionists. Furthermore, they found that the traditional training and competition diet consists of a limited variety of foods, which makes for a dull diet, and results in players adding other foods of inadequate nutritional value. Finally, the eating practices of these players were mainly linked to dietary habits acquired during childhood, which can influence food preferences and eating practices in adulthood. This qualitative information is very important, as it highlights some of the correlates of eating practice that are difficult to identify using a quantitative approach, but should be considered when designing and implementing effective nutritional intervention programs.

## 5. Correlates of the Eating Practice and Nutritional Intake of Soccer Players

The many correlates of eating practice offer a plethora of potential avenues through which food selection and nutrient intake can be modified [91]. However, many of these factors are difficult to quantify and interact with other variables. Add to this a highly competitive sport environment, and the picture becomes even more complex. In the following sections, we will analyze the little information available on the influence of some of these correlates on eating practices in soccer players.

### 5.1. Food Preferences

Food likes and dislikes are strong correlates of eating practice [91] and have been described as predictors of food selection and even of nutrient intake in several studies in different populations [92,93,94]. There is general consensus that understanding food preferences is essential to plan effective nutrition education programs [90,92,95]. However, to the best of our knowledge, we are the only group to have analyzed this parameter in soccer players [84], and we found no evidence of a relationship between food preferences and food and nutritional intake.

To analyze food preferences, we used a questionnaire that included 15 food groups, which players assessed were asked to rank from 1 to 15 according to their preference. Three categories were established according to the ranking of food preferences: “Like”, for food groups ranked between 1 and 5; “Indifferent”, for those ranked between 6 and 10; and “Dislike”, for all food groups ranked 11–15. The main preferences were meat, poultry and derivatives, and pasta, while vegetables and fish were designated as the main dislikes. Next, food intake was recorded using a food frequency questionnaire. Foods were classified into 15 groups (matching as close as possible those of the food preferences questionnaire) and the number of standard portions of each food group ingested per day was calculated. Finally, we compared the daily number of standard portions consumed from each of the different preference groups. This revealed no statistical differences between groups, indicating that food preferences did not influence food intake. Energy and macronutrient intake was recorded using the weighed food record method, and the relative contribution of different food groups to the total daily energy and macronutrient intake was determined. Again, we found no significant correlation between food preferences and the intake of energy and macronutrients or the contribution of food groups to total intake.

The players assessed were adolescents living in their family environment. In this situation, the possibility of selecting foods according to individual preference is limited, as meals are usually under the supervision of other family members, who select the daily menu based on several factors (e.g., attitudes towards health and nutrition, food cost, ease of preparation) distinct from the player’s food preferences. Thus, of the wide range of factors that influence food selection and nutrient intake, food preferences are not a critical determinant for soccer players. The influence of the family environment appears to be stronger and should be borne in mind when designing and implementing nutritional interventions for soccer players, especially for adolescents. It would be necessary to confirm these results with adult players.

### 5.2. Playing Position

It has frequently been reported that the spontaneous nutritional intake of athletes is related to the physiological and metabolic demands of their sport [96,97,98,99]. Many authors have described position-related differences in the performance capabilities and physiological and anthropometric characteristics of soccer players. This is related to the particular activity profile of each playing position, which affects the proportion of aerobic and anaerobic energy production [1,5,7,12,14,19,20,21,22,23,24,25,26,27,28,29,30,100]. While the differences in the physiological demands between playing positions are much less marked than those between different disciplines, they are nonetheless significant. In their guidelines for daily CHO intake for soccer players, Burke and coworkers differentiate between “mobile” and “less mobile” players based on the nutritional demands associated with their playing position [34]. However, there is little information available about the food and nutrient intake of soccer players relative to their field position (Table 4) [43,101,102,103]. Moreover, some of these articles do not provide information for the entire team, and thus are not included in Table 1. No information on female players is available.

Not all the authors addressing this question have reported significant differences in nutrient intake associated with the physiological and metabolic demands of specific playing positions, although this may be explained by the many methodological differences between studies. Besides, most authors provide no information about the training programs. Therefore, it is possible that the lack of differences might be due to very likely similar programs, with no marked positional differences in the type, duration, and intensity of the activities involved.

In our opinion, before assessing positional differences in the nutrient intake of soccer players, it is essential to corroborate the positional differences in the performance capabilities and the physiological and anthropometric characteristics of the players assessed. However, this has not been verified in most studies. Furthermore, a study by Innocencio da Silva Gomes and colleagues [102] assessed a cohort of disabled soccer players, whose amputations impose greater locomotory costs and hence different physiological demands to those of able players. Moreover, these players’ anthropometric characteristics, although corrected for the amputation, did not coincide with the positional profile described in the literature.

**Table 4:** Studies examining the energy and macronutrient intake of soccer players according to their playing position on the field.

Nutrients	Innocencio da Silva Gomes *et al.* 2006 [102] *			Conejos *et al.* 2011 [103] ^b^				Iglesias-Gutiérrez *et al.* 2012 [43]						Imamura *et al.* 2013 [101] ^1^	
	Full-Backs (*n* = 3)	Midfielders (*n* = 2)	Forwards (*n* = 6)	Goalkeepers (*n* = 3)	Defenders (*n* = 6)	Midfielders (*n* = 6)	Forwards (*n* = 7)	Goalkeepers (*n* = 12)	Full-Backs (*n* = 12)	Centre-Backs (*n* = 15)	Midfielders (*n* = 24)	Wingers (*n* = 12)	Forwards (*n* = 12)	Defenders (*n* = 16)	Offenders (*n* = 15)
Energy intake (kcal)	3384 ± 1128	4294 ± 651	3832 ± 1245	2915.9 ± 1099.4	3537.3 ± 621.4	3346.1 ± 1481.8	3035.4 ± 693.1	2600 ± 641	2766 ± 452	2771 ± 582	2855 ± 475	2881 ± 385	2779 ± 659	2996 ± 949	2815 ± 716
*Proteins*															
g	193.7 ± 6.2	231.9 ± 30.0	200.1 ± 63.1	142.8 ± 100.1	144.5 ± 19.9	144.8 ± 56.9	138.7 ± 27.5	115 ± 29	117 ± 24	120 ± 21	117 ± 21	118 ± 22	115 ± 27	84.4 ± 32.0	76.3 ± 21.3
g/kg BM	3.0 ± 1.1	3.1 ± 0.8	3.2 ± 0.9	-	-	-	-	1.5 ± 0.4	1.7 ± 0.4	1.7 ± 0.4	1.6 ± 0.3	1.7 ± 0.4	1.6 ± 0.3	-	-
% energy	23 ^a^	21 ^a^	21 ^a^	17.9	16.9	17.8	18.4	18 ± 2	17 ± 2	17 ± 2	16 ± 3	16 ± 2	17 ± 2	-	-
*CHO*															
g	401.8 ± 135.4	515.5 ± 72.5	505.3 ± 156.3	320.3 ± 11.9	419.1 ± 98.3	382.1 ± 187.2	342.5 ± 92.9	304 ± 35	346 ± 58	313 ± 65	352 ± 72	352 ± 34	342 ± 92	456.3 ± 145.0	409.9 ± 98.1
g/kg BM	6.2 ± 2.4	7.0 ± 1.9	8.1 ± 2.2	-	-	-	-	3.9 ± 1.0	4.9 ± 1.0	4.3 ± 1.1	4.9 ± 1.3	4.9 ± 0.8	4.6 ± 1.2	-	-
% energy	48 ^a^	49 ^a^	53 ^a^	17.9	44.2	48.4	44.8	44 ± 3	47 ± 5	42 ± 4	46 ± 6	46 ± 4	46 ± 6	-	-
*Lipids*															
g	-	-	-	109.8 ± 45.3	124.5 ± 36.1	131.6 ± 62.6	120.3 ± 59.6	111 ± 32	110 ± 28	124 ± 34	118 ± 32	121 ± 21	115 ± 35	-	-
% energy	29 ^a^	30 ^a^	26 ^a^	34.9	32.2	35.6	34.7	38 ± 4	36 ± 5	40 ± 4	37 ± 6	38 ± 3	37 ± 6	-	-
MUFA	-	-	-	13.7	13.1	14.4	10.7	-	-	-	-	-	-	-	-
PUFA	-	-	-	6.9	5.9	5.5	8.1	-	-	-	-	-	-	17.7 ± 5.8	20.4 ± 5.2
SFA	-	-	-	10.8	10.5	12.7	11.9	-	-	-	-	-	-	44.7 ± 12.9	49.3 ± 14.3

BM: Body mass; CHO: carbohydrates; MUFA: Monounsaturated fatty acids; PUFA: Polyunsaturated fatty acids; SFA: Saturated fatty acids. Unless stated otherwise, data are presented as Mean ± Standard deviation: ^a^ Mean; ^b^ No information was provided for the authors about how the results are reported. Unless stated otherwise, MUFA, PUFA, and SFA intake are referred as percentage of total energy intake: 1 intakes expressed as g/day. * One goalkeeper was also analyzed but not included in the results. All data about energy intake have been converted to kcal. Blank cells indicate that this information was not provided by the authors.

In the studies reviewed here, players are divided into several categories based on field position. Most of these studies only apply a horizontal subdivision (goalkeepers, defenders, midfielders, and forwards). However, a vertical subdivision (players in the wings and players in the center) is necessary to more clearly indicate the positional differences in the physiological demands, activity profiles and physical characteristics of soccer players [22]. Thus, some of the groups described are not sufficiently specific, and include players with markedly different positional profiles.

Furthermore, evaluation of the nutritional intake of players in different field positions requires a large number of participants, as considerable variations in many parameters have been found between players for a given field position [5,20]. The aforementioned Innocencio da Silva Gomes study assessed only 11 players divided into three different positional groups [102], while that of Conejos and coworkers featured only three to seven players per playing position [103]; these small sample sizes diminish the statistical power of the analysis.

The methodological characteristics mentioned above influence nutrient intake, as well as nutrient needs and food selection, which greatly complicates comparison between studies. Despite these difficulties, some differences in nutrient intake between playing positions have been reported. We have reported a higher spontaneous CHO intake in players whose field positions demand a higher proportion of aerobic energy production and found that the contribution to daily energy intake from cereals and derivatives, and potatoes was higher in these players [43]. Differences in macronutrient intake, albeit unrelated to the specific demands of field position, were also reported by Conejos and coworkers [103]. Those authors also described position-related differences in vitamin and mineral intake, although no evident position-related profile was observed.

These differences in food and nutrient intake demonstrate that mean group values can be affected by positional profile, and should be taken into account in future studies of the nutritional intake of team sport athletes. Furthermore, due to the little information on this topic, only available on men, it is worthy of properly studying in order to inform individualized recommendations for players by position.

### 5.3. Nutritional Intake on Match Days

Many soccer players and technical staff traditionally consider pre- and post-game meals as a nutritional priority [34,104]. Menu planning has thus received much attention as the cornerstone of a successful nutritional strategy for competition [105]. However, in many cases it remains unclear whether nutritional recommendations for pre- and post-game meals are applied in practice [104,105], especially for away matches, when set menus are usually offered to the players. Furthermore, it might be thought that nutritional adequacy would be higher, better distributed across meals, with more attention to post-workout recovery nutrition, *etc.*, when meals and meal plans are provided compared to players who self-select their own food. It is worth exploring whether it could constitute an alternative to the implementation of nutrition education programs. Thus, an interesting question is: to what extent can eating practices on match days be attributed to the set menus offered, the players’ ability to select food, or a combination of these two factors?

Several authors have suggested that players show a specific and differentiated dietary behavior for match days, which could influence energy and nutrient intake. Holway and Spriet suggested that stress, travel, and match schedules can alter the eating habits of team sport athletes, causing them to eat less on match days than training days, resulting in inadequate levels of energy and macronutrient intake for competition and recovery [104]. However, those authors did not consider the influence of set menus. By contrast, Burke and colleagues claimed that soccer players traditionally attach more importance to pre- and post-game meals than to daily diet, suggesting that nutrient intake is closer to optimal on match days due to the application of a specific dietary regimen [34]. While these contradictory ideas are both interesting, the respective authors provide no data with which to support their claims.

Furthermore, there is little or no information available on the specific nutrient intake of soccer players on match days, and the sole study that has assessed this parameter has significant limitations [106]. In that study, players were assessed only on one match day, using a 24-h recall, which gives little validity to the results obtained. Moreover, the authors did not indicate if it was a home or an away match, nor do they mention whether the players were provided with set menus. Interestingly, energy and CHO intake were higher on the match day as compared with one training day. Nonetheless, further research is needed to better understand the players’ eating practices in this specific scenario in order to develop evidence-based guidelines for the optimization of menu design and nutrient intake in the match day diets of soccer players.

## 6. Conclusions

There is a virtually unanimous agreement on the inadequate nutrient intake of male and female soccer players, and the need for the design and implementation of nutrition education programs to address this problem. Currently, however, the majority of studies available are small, limited in scope, cross-sectional, and the conclusions derived should be viewed with caution. Furthermore, the information available on the factors that influence the eating practices of soccer players is insufficient to develop a successful intervention program, underscoring the need for further research in this area, as well as for more resources for this kind of research on a larger scale.

Improving the eating practices of these athletes will help them to optimize performance and to promote healthy eating habits that will provide benefits well beyond the end of their careers.
